# The Diagnostic Value of Both Troponin T and Creatinine Kinase Isoenzyme (CK-MB) in Detecting Combined Renal and Myocardial Injuries in Asphyxiated Infants

**DOI:** 10.1371/journal.pone.0091338

**Published:** 2014-03-13

**Authors:** Wilson E. Sadoh, Charles O. Eregie, Damian U. Nwaneri, Ayebo E. Sadoh

**Affiliations:** 1 Department of Child Health, University of Benin/University of Benin Teaching Hospital, Benin City, Nigeria; 2 Institute of Child Health, University of Benin/University of Benin Teaching Hospital, Benin City, Nigeria; CNR, Italy

## Abstract

**Background:**

Troponin T (cTnT) and Creatinine Kinase Isoenzyme (CK-MB) are both markers of myocardial injuries. However, CK-MB is also elevated in acute kidney injury.

**Objective:**

The diagnostic value of both cTnT and cardiac CK-MB in combined myocardial and acute kidney injuries (AKI) in asphyxiated neonates was evaluated.

**Method:**

40 asphyxiated infants and 40 non-asphyxiated controls were consecutively recruited. Serum levels of cTnT, CK-MB and creatinine were measured. Myocardial injury and AKI were defined as cTnT >95^th^ percentile of the control and serum creatinine >1.0 mg/dl respectively.

**Results:**

Of the 40 subjects, 9 (22.50%), 8 (20.00%) and 4 (10.00%) had myocardial injury, AKI and combined AKI and myocardial injuries respectively. The mean cTnT and CK-MB values were highest in infants with combined AKI and myocardial injuries. The Mean cTnT in infants with AKI, myocardial injury and combined AKI and myocardial injuries were 0.010±0.0007 ng/ml, 0.067±0.040 ng/ml and 0.084±0.067 ng/ml respectively, p = 0.006. The mean CK-MB in infants with AKI, myocardial injury and combined AKI and myocardial injuries were 2.78±0.22 ng/ml, 1.28±0.11 ng/ml and 4.58±0.52 ng/ml respectively, p = <0.0001.

**Conclusion:**

In severe perinatal asphyxia, renal and myocardial injuries could co-exist. Elevated cTnT signifies the presence of myocardial injury. Elevated CK-MB indicates either myocardial injury, AKI or both. Therefore renal injury should be excluded in asphyxiated infants with elevated CK-MB.

## Introduction

Perinatal asphyxia is a major contributor to neonatal morbidity and mortality especially in developing countries where facilities for neonatal care are limited [1], [2]. Severe birth asphyxia is reported to contribute 22.0% of neonatal morbidity and 20.0% of neonatal mortality of hospital newborn admissions in Nigeria [3], [4]. Perinatal asphyxia is an antepartum/post-partum hypoxic ischaemic injury to the foetus/baby which compromises oxygen delivery and removal of carbon dioxide from the fetus. There is a redistribution of cardiac output with a larger amount to the coronary, adrenal and cerebral circulations while reducing output to the skin, intestines and the kidney in the so called dive reflex. [5] The organs with the compromised blood supply are thus susceptible to injury resulting in a multi-organ illness. [6] Cerebral hypoxic ischaemic injury is the most important consequence of perinatal asphyxia.

Perinatal asphyxia is detected by poor APGAR scores and the low arterial cord P^H^ in the newborn. [7], [8] APGAR scores is a reflection of the state of the newborn and the reaction to certain stimulus. It is a tool that facilitates rapid assessment of the presence and severity of perinatal asphyxia in the newborn. It is however not very sensitive, being affected by factors such as maternal sedation and prematurity. [7] The P^H^ of the newborn is more sensitive in detecting perinatal asphyxia. [8] The combination of APGAR score and P^H^ determination enhances the detection of perinatal asphyxia. In most resource limited settings, facilities for P^H^ determination is often lacking and APGAR scores remains the only means of detecting perinatal asphyxia.

Myocardial ischaemia and infarction are not uncommon in infants with perinatal asphyxia. Biochemical markers along with electrocardiographic and echocardiographic features have been used to identify infants with myocardial complications. Cardiac Troponin T (cTnT) is a good marker of myocardial ischaemia/infarction in infants with perinatal asphyxia [9]. It is more sensitive and specific than cardiac creatinine phosphokinase isoenzyme (CK-MB) also used in identifying infants with myocardial involvement [9], [10]. Unlike CK-MB, cTnT is not affected by gestational age, mode of delivery and other related factors [10], [11]. This is more so when a third generation assay is used in cTnT assay. The CK-MB is also elevated in conditions of skeletal muscle damage, resulting in false positive CK-MB test. [9] In an attempt to define the value above which cTnT can be said to be elevated in newborn, Clark et al [12] used the 95^th^ percentile of values obtained in cord blood of normal neonates.

Acute kidney injury (AKI) is also an important complication of perinatal asphyxia [13]. AKI affects about 70% of infants with severe birth asphyxia [14]. Evidence of renal insult includes electrolyte abnormalities specifically hyperkalaemia and hyponatraemia. Elevated creatinine, though having its drawback, is for now the standard serum marker in renal insult following acute renal shut down [15]. CK-MB, though less sensitive than cTnT in identifying myocardial injury, is elevated following renal injury [16]. Thus, in perinatal asphyxia, CK-MB may be elevated not only when there is myocardial injury but also when there is associated renal injury or when only renal injury occurs.

This study set out to evaluate the levels of CK-MB and cTnT in perinatally asphyxiated infants to identify those with renal, myocardial and combined renal and myocardial injuries.

## Materials and Methods

### Ethics Statements

Ethical approval for the study was obtained from the University of Benin Teaching Hospital ethical Committee. Written informed consents were obtained from the mothers of the infants.

### Recruitment of Subjects and Controls

Consecutive babies delivered at the University of Benin Teaching Hospital, Benin City, Nigeria who had moderate to severe perinatal asphyxia were recruited for the study. Asphyxia was determined by the use of APGAR scores at 1 and 5 minutes. Score of ≤3 in the first minute and <5 in the fifth minute were taken as severe birth asphyxia while scores of 4 and 5 in the first minute were taken as moderate birth asphyxia [7]. Forty newborns with APGAR scores ≥7 were also recruited as control. The control group was matched by birth weight. The study was conducted between January and December 2009.

The relevant information on the babies obtained were age at recruitment, gender, gestational age determined by the last menstrual period and the gestational maturity was determined by Eregie and Muogbo chart [17]. Preterm babies were those born at <37 completed weeks while the term babies were born at ≥37 completed weeks. The birth weight was measured with an infant weighing scale.

### Laboratory Tests

Two milliliters of blood was obtained from the infants via an aseptic procedure for the determination of cTnT and CKMB. The blood was spun and the plasma was decanted into a plain universal bottle and kept in a freezer at −40° centigrade until it was ready for analysis. The cTnT and CK-MB were determined by the electrochemiluminescense immunoassay method with Elecsys 2010 Analyser manufactured by Roche Hitachi, Germany 2004. The lower limits of measurement of the assay were 0.01 ng/ml and 0.01 ng/ml respectively for cTnT and CK-MB. The troponin T test kit number was 159300–01(04491815) while the CK-MB test kit number was 159025-01 (11821598 332). They were manufactured by Roche Diagnostics Mannheim, Germany in 2009. All the assays were done by a laboratory scientist according to the manufacturer’s specification. Another blood sample obtained after 24 hours of life was analyzed for serum creatinine and electrolyte profile. This was to ensure the values were those of the baby and not a reflection of the mothers since it takes 24 hours for creatinine levels to rise [15]. Creatinine levels were considered elevated if >1.0 mg/dl [15]. Hypokalaemia and hyperkalaemia were defined using standard cut-offs. A Case of myocardial injury was defined as an infant with elevated serum cTnT >95 percentile of the controls while renal impairment was defined as infants with serum creatinine >1.0 mg/dl.

### Statistical Analysis

All the data were coded and entered into SPSS version 16.0 and analysis was done with this tool. Simple proportions were expressed in percentages, while means and standard deviations (SD) were computed for continuous variables. Differences in proportions were determined using χ^2^ or Fisher’s Exact tests while the comparison of means of variables for significant difference was done with Student t-test or one-way ANOVA. Statistical significance was set at P value <0.05 at 95% confidence interval.

## Results

Forty neonates with moderate to severe asphyxia were recruited for the study. The mean age was 33.3±27.8 hours. There were 17 (42.5%) males and 23 (58.5%) females while 32 (80.0%) were severely asphyxiated. Most of the neonates were term 29 (72.5%), while 11 (27.5%) were preterm. The mean gestational age and mean birth weight were 39.1±3.1 weeks and 2.95±0.69 kg respectively.

The mean cTnT and CK-MB values of the subjects were 0.03±0.04 (range; 0.01–0.16) ng/ml and 2.3±2.5 (range; 0.03–11.97) ng/ml respectively. The mean cTnT of controls was 0.01±0.0006 (range 0.01–0.013) ng/ml while the mean CK-MB of the control was 0.339±0.49 (range 0.10–02.40) ng/ml. The differences in mean cTnT and CK-MB between the subjects and controls were statistically significant; p = 0.0022 and <0.0001 respectively for cTnT and CK-MB. The 95^th^ percentile of the cTnT and CK-MB of the controls were 0.012 and 2.14 ng/ml respectively. Serum creatinine was available for 34(85.0%) of the 40 subjects. The mean serum creatinine was 1.01±0.25 (range; 0.70–1.70) mg/dl. Hyperkalaemia and hypokalaemia were present in 3 (8.8%) and 4 (11.8%) of the 34 subjects respectively, while potassium level was normal in 27 (79.4%) of the subjects.

Of the 40 neonates, 13 (32.50%) were adjudged to have myocardial injury (cTnT values ≥0.012 ng/ml), their mean cTnT level was 0.072±0.047 ng/ml. This was significantly higher than the value obtained in infants without myocardial injury (cTnT <0.012 ng/ml) which was 0.01±0.00037 ng/ml, P = <0.0001 (95% CI = −0.08 to –0.04). Of the 34 infants with creatinine values, 12 (35.3%) had AKI (elevated creatinine values >1.0 mg/dl). Their mean serum creatinine 1.28±0.21 mg/dl was significantly higher than the 0.86±0.10 obtained in infants without AKI (creatinine values <1.0 mg/dl), P = <0.0001 (95% CI = −0.53 to –0.31). There were 4 (40.0%) cases of combined renal and myocardial injuries while 19 infants had neither elevated cTnT nor CK-MB. Table 1 shows the mean values of the infants with elevated cTnT, creatine and those with normal values.

**Table 1:** The mean cTnT and CKMB levels in infants with renal, myocardial, combined renal and myocardial and no injury.

	Organ impairment				
Tests	*Combined	Renal only	Myocardial only	None	P value
Troponin T	0.084±0.067	0.010±0.0007	0.067±0.040	–	0.006
CK-MB	4.58±0.52	2.78±0.22	1.28±0.11	2.02±0.21	<0.0001

*Combined = combined renal and myocardial damage.

The mean and standard deviation of the troponin T value for infants in the ‘none’ column could not be computed by the statistical package because the troponin t values for the infants were too low.

The mean CK-MB values of the infants with AKI, 3.38±0.33 ng/ml was significantly higher than the value observed in those without AKI 2.00±0.2 ng/ml, P = <0.0001 (95% CI = −1.56 to −1.20). The mean cTnT level in infants with AKI 0.035±0.050 was not significantly higher than in those without AKI, P = 0.39 (95% CI = 0.04 to 0.016. There was no significant difference in mean CK-MB between infants with myocardial injury 2.30±0.31 ng/ml and those without myocardial injury 2.25±021 ng/ml, P = 0.55 (95% CI = −0.22 to 0.12). The mean cTnT and CK-MB values of combined cases of AKI and myocardial injuries (0.084±0.067 ng/ml and 4.58±0.52 ng/ml respectively), were significantly higher than the mean values obtained in the cases of isolated AKI and myocardial injuries, P = <0.0001 and 0.006 respectively (Table 1).

In this study, 8 (20.0%) newborns died while 32 (80.0%) were discharged home. Of the 4 cases of combined AKI and myocardial infarction 2 (50.0%) died while 20.0% and 11.1% respectively died amongst the infants with AKI and myocardial injury only. The differences were not significant, P = 0.39 (Table 2).

**Table 2:** Mortality in asphyxiated infants with renal, myocardial, combined renal and myocardial injuries.

Type of injury	Total number	Number of mortality	Percentage
Combined renal and myocardial	4	2	50.0
Renal only	8	2	20.0
Cardiac only	9	1	11.1
None injured	19	3	15.8

P = 0.39.

## Discussion

In this study, the levels of cTnT and CK-MB were evaluated in asphyxiated infants to identify those with AKI, myocardial injury and combined injuries. Both enzymes were elevated in the presence of myocardial injury; however CK-MB was also elevated in the presence of renal injury. The cases of combined AKI and myocardial insult had CK-MB and cTnT that were significantly more elevated than observed in the cases of isolated AKI or myocardial insult. The elevated values may have been due to the severity of the perinatal asphyxia, which was severe enough to have affected multiple organs including the heart and kidneys. All the cases of combined insults in this study had severe perinatal asphyxia. Previous studies have indicated that the severity of asphyxia as demonstrated by increasing P^H^ and decreasing APGAR scores correlates with the increasing levels of cTnT [18],[19]. It is opined from this study, that grossly elevated values of CK-MB or cTnT may indicate more than myocardial injury when found in infants being evaluated for perinatal asphyxia. The possibility of renal insult should be sought by evaluating for possible renal injury.

In this study, AKI was detected using elevated creatinine value which is deemed not very reliable [20]. However, there are other proposed markers of AKI, which include cystacin C, neutrophil gelatinase-assocaited lipocalin and kidney injury molecule [21]. These markers are not being used currently in clinical practice. The creatinine values are more reliable if the measurement is done beyond 24 hours of life, so they don’t reflect the maternal values as it takes 24 hours for levels of creatinine to rise in renal injury [15]. Creatinine evaluation is commonly available in resource-poor settings and was the marker used in this study. Other renal function derangements such as potassium and sodium abnormalities may not occur in the face of elevated creatinine values as demonstrated in a previous study [15]. This indicates the unreliability of using isolated electrolytes derangements as possible evidence of renal injury. This non-constant changes may explain the insignificant difference in CK-MB values of infants with potassium abnormalities (hyperkalaemia and hypokalaemia) and those with normal potassium.

The mean cTnT value of patients with myocardial injury was significantly elevated compared to those without myocardial injury. This is in keeping with findings from previous studies [9], [18], [19]. In this study, however, CK-MB levels in infants with myocardial injury were not significantly higher than in infants without myocardial injury. This has been previously reported [22], [23]. This is due to the higher sensitivity of the cTnT test compared with CK-MB in detecting myocardial injury as shown by a number of studies [22], [23].

CK-MB was significantly higher in infants with AKI compared to infants without AKI. This may be due to the close relationship that CK-MB has with creatinine phosphokinase and creatinine which are elevated in renal damage. CK-MB is the myocardial fraction of creatinine phosphokinase. The clinical implication of elevated CK-MB in the setting of perinatal asphyxia is possible renal injury. Such a finding should encourage further evaluation of renal functions in the newborn. We note, however, that the small numbers of infants in the study limit the inference drawn from the findings.

The mortality profile in this study shows a high mortality among children with combined renal and myocardial injuries compared to cases with isolated myocardial injury and AKI. This further buttresses the influence of the severity of asphyxia on the combined cases.

In conclusion, in severe perinatal asphyxia, renal and myocardial injuries may occur and could be shown by grossly elevated CK-MB and cTnT values. The finding of elevated CK-MB in perinatally asphyxiated infant may not be attributable to myocardial infarction alone. The possibility of renal injury as a cause of elevated CK-MB should explored.
